# Ambulatory blood pressure profiles in a subset of HIV-positive patients pre and post antiretroviral therapy

**DOI:** 10.5830/CVJA-2014-029

**Published:** 2014

**Authors:** Megan Borkum, Athlet Alfred, Nicola Wearne, Brian Rayner, Joel A Dave, Naomi S Levitt

**Affiliations:** Department of Medicine, University of Cape Town, Cape Town, South Africa; Department of Medicine, University of Cape Town, Cape Town, South Africa; Department of Nephrology and Hypertension, University of Cape Town, Cape Town, South Africa; Department of Nephrology and Hypertension, University of Cape Town, Cape Town, South Africa; Division of Diabetic Medicine and Endocrinology, University of Cape Town, Cape Town, South Africa; Division of Diabetic Medicine and Endocrinology, University of Cape Town, Cape Town, South Africa

**Keywords:** human immunodeficiency virus, antiretroviral therapy, microalbuminuria, chronic kidney disease, ambulatory blood pressure, non-dipping

## Abstract

**Objectives:**

Human immunodeficiency virus (HIV) and antiretroviral therapy (ART) are associated with renal disease and increased cardiovascular risk. The relationship between HIV and ambulatory blood pressure (ABP) non-dipping status, a risk factor for cardiovascular events and target-organ damage, has never been assessed in South Africa. Study objectives were to establish the prevalence of chronic kidney disease, and assess the ABP profile in asymptomatic HIV-positive clinic out-patients.

**Methods:**

This was a prospective cohort study. Office blood pressure (BP), urinary microalbumin–creatinine ratio, urine dipsticks, serum creatinine and estimated glomerular filtration rate (eGFR) were measured at baseline and six months after ART initiation. A subset of HIV-positive subjects and an HIV-negative control group underwent 24-hour ABP monitoring.

**Results:**

No patient had an eGFR < 60 ml/min, three patients (4.7%) had microalbuminuria and one had macroalbuminuria. Mean office systolic BP was 111 ± 14 mmHg at baseline and increased by 5 mmHg to 116 ± 14 mmHg (*p* = 0.05) at six months. This increase was not confirmed by ABP monitoring. In the HIV-positive and -negative patients, the prevalences of non-dipping were 80 and 52.9%, respectively (*p* = 0.05, odds ratio = 3.56, 95% CI: 0.96–13.13). No relationship between dipping status and ART usage was found.

**Conclusion:**

The prevalence of chronic kidney disease (CKD) was lower than anticipated. HIV infection was associated with an ambulatory non-dipping status, which suggests an underlying dysregulation of the cardiovascular system. In the short term, ART does not seem to improve loss of circadian rhythm.

## Abstract

South Africa has 5.6 million people living with HIV/AIDS and has the largest antiretroviral therapy (ART) programme globally, with more than two million people accessing ART.^1^ Although ART has significantly decreased the mortality rate from HIV infection, these individuals are now living longer and are at risk of developing metabolic (dyslipidaemia, lipodystrophy, dysglycaemia), cardiovascular and renal complications from ART and chronic exposure to HIV infection.^2^-^7^

Chronic HIV and ART are associated with increased risk of developing hypertension.^8^ In studies of HIV-positive patients in high-income countries, hypertension prevalence ranges from 13 to 34%.^9^,^10^ However, data from low- and middle-income countries remain sparse.

Nocturnal blood pressure (BP) is superior to daytime or office BP as a predictor of cardiovascular disease.^11^ Non-dipping is defined as an abnormal diurnal rhythm manifested by a blunted nocturnal decline in systolic BP (SBP).^11^ It is associated with more severe hypertensive target-organ damage (left ventricular hypertrophy, microalbuminuria and cerebrovascular disease) and is also a predictor of increased cardiovascular risk, both in hypertensive and normotensive populations.^11^

Studies from high-income countries have shown an increased prevalence of non-dipping with HIV infection.^9^,^12^ However, the participants in these studies were largely white, middle-aged males. Since the majority of subjects with HIV infection in sub-Saharan Africa are young black females, it is not known whether the same relationship between dipping status and HIV infection would be found. In addition, there are data showing that black HIV-negative individuals have less nocturnal dipping compared to their white counterparts.^5^,^13^,^14^

Therefore, the aims of this study were to document the prevalence of chronic kidney disease (CKD) and hypertension at baseline (ART naïve) in a healthy HIV-positive cohort, and to assess changes in these parameters after six months on ART. The characteristics of ambulatory blood pressure (ABP) in a subset of patients were to be recorded and compared to a control group of HIV-negative patients.

## Methods

A longitudinal, prospective cohort study was conducted. It was approved by the Research Ethics Committee of the Faculty of Health Sciences of the University of Cape Town. Before participating in the study, procedures and risks were explained to the patients, who gave written informed consent to take part in the study.

**Fig. 1.**
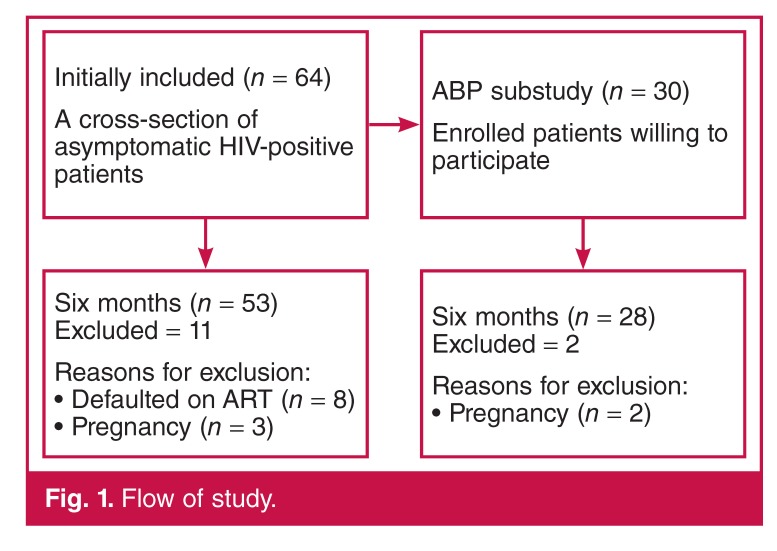
Flow of study.

This study formed part of an as yet unpublished larger longitudinal study, investigating the metabolic complications of ART in an HIV-positive population, at an HIV clinic in a community health centre in Cape Town, South Africa. All patients recruited for the parent study over a six-month period were enrolled into this study (Fig. 1).

The following measurements were done at baseline and repeated at six months: urine dipstick (AccuBioTech Co. Ltd, Beijing, China), office BP, serum creatinine (umol/l), spot urine microalbumin–creatinine ratio (mg/mmol), and estimated glomerular filtration rate (eGFR) (ml/min/1.73 m^2^). Three office BP readings were performed on the right arm with the patient in a seated position using a mercury barometer in accordance with the South African hypertension guidelines.^15^

A urinary albumin–creatinine ratio between 3 and 30 mg/mmol was identified as microalbuminuria and a level greater than this as macroalbuminuria.^16^ eGFR was estimated using the four-variable Modification of Diet in Renal Disease (MDRD) equation, which accounts for the gender, age, creatinine level and race of a patient.^17^ Clinical guidelines from the Kidney Disease: Improving Global Outcomes (KDIGO) work group were used to categorise CKD.^18^

After the baseline measurements, all patients were started on ART (Table 1). The treatment regimen used depended on the date of enrolment into the study. Initially patients were prescribed stavudine (D4T), lamivudine (3TC) and efavirenz (EFV) according to the previous national guidelines, but later tenofovir (TDF) replaced D4T.^19^,^20^

**Table 1:** Patient characteristics and demographics

	*Baseline (n = 64)*	*Six months (n = 53)*	*ABPM group at baseline (n = 30)*	*ABPM group at six months (n = 28)*	*Controls (n = 17)*
Age (years) mean ± SD	33 ± 7	33 ± 7	32 ± 8	32 ± 8	31 ± 9
Men (%)	23	23	37	36	40
Women (%)	77	77	63	64	60
BMI (kg/m^2^) mean ± SD	24.8 ± 5.4	25.7 ± 5.2	24.6 ± 5.2	24.8 ± 5.4	24.0 ± 4.8
Men	22.5 ± 4.6	23.1 ± 4.8	22.9 ± 5.0	22.7 ± 5.3	22.8 ± 5.1
Women	25.5 ± 5.4	26.9 ± 5.6	25.4 ± 4.9	25.8 ± 5.4	25.2 ± 4.8
CD_4_ (cells/mm^3^) median	239	359	242	361	N/A
(IQR)	(169–322)	(231–411)	(165–330)	(240–406)	
ART regimen (%)					
Curren	–	67	–	72	–
Earlier	–	29	–	26	–
Other	–	4	–	2	–

All enrolled patients were invited to participate in the ABP substudy. Consenting individuals underwent ABP monitoring prior to and after the initiation of ART. A control group of confirmed serologically HIV-negative patients formed the control group of another study from our institution investigating HIV-associated dementia.^21^ They were originally recruited by trained fieldworkers from a community primary healthcare clinic in Cape Town.

Seventeen individuals from a list of 32 contacted telephonically were available to participate. They were equally matched for age, body mass index (BMI) and socio-economic background. Patients were excluded if they had underlying hypertension, diabetes mellitus, ischaemic heart disease, concurrent illness or any condition affecting BP (i.e. pregnancy or renal disease).

ABP monitoring was set up by a trained nurse on a weekday, with an oscillometric device (SpaceLabs Medical Inc, WA, USA). BP and heart rate were recorded every 20 minutes during the day (06:00 to 22:00) and every 30 minutes at night (22:00 to 06:00). Hypertension was defined as a SBP ≥ 140 mmHg or diastolic BP (DBP) ≥ 90 mmHg, in accordance with the South African hypertension guidelines 2011.^15^ Non-dipping was defined as a nocturnal reduction of SBP ≤ 10%.^22^

## Statistical analyses

Statistical analyses were performed using STATA statistical software, version 11.0 (STATA Corporation, College Station, Texas, USA). Mean ± standard deviation was used for normally distributed data and median plus interquartile ranges for skewed data. Continuous and categorical variables were compared using chi-square, Student’s *t*-test or Pearson’s χ^2^ as appropriate. All *p*-values were considered significant at *p* ≤ 0.05.

## Results

Sixty-four patients were entered into the study, with baseline characteristics as shown in Table 1. All were black South Africans, mean age 33 ± 7 years, and 77% were female. Eleven patients were excluded on follow up [defaulted on treatment (*n* = 8), pregnant (*n* = 3)].

Mean BMI was 24.8 ± 5.4 kg/m^2^ and increased to 25.7 ± 5.2 kg/m^2^ after six months on ART (*p* = 0.39). At baseline, median CD_4_ count was 239 (169–322) cells/mm^3^, and after six months of ART the CD_4_ count increased to 359 (231–411) cells/mm^3^. All patients had suppressed viral loads.

Thirty patients agreed to participate in the ABP substudy. Two were excluded at six months due to pregnancy.

There were no significant differences between those who underwent ABP monitoring and those who did not, according to age, gender, ethnicity, BMI, CD4 count, ART status or office BP. Baseline demographics were similar in the HIV-negative control group except there were more males in this group compared with the HIV-positive cohort. However, there was still a greater percentage of females than males in the control group (Table 1).

At baseline, 13 patients (20%) had an eGFR of 60–89 ml/min/1.73 m^2^ (GFR category G2). No patient had an eGFR < 60 ml/min/1.73 m^2^ (Table 2). Microalbuminuria was present in three of the 64 patients (4.7%) and only one patient (1.6%) had macroalbuminuria. At the end of six months, microalbuminuria persisted in the three patients and developed in two new cases. In the patient who had initially had macroalbuminuria, it resolved on follow-up sampling. No patient had a change in eGFR over the study period.

**Table 2:** BP and renal parameters at baseline and six months

	*Baseline (n = 64)*	*Six months (n = 53)*	p*-value*
MDRD eGFR (ml/min/1.73 m^2^)	109 ± 23	107 ± 22	0.66
≥ 90, *n* (%)	51 (80)	51 (80)	–
60–89, (%)	13 (20)	13 (20)	–
< 60, *n* (%)	0 (0)	0 (0)	–
Office systolic BP (mmHg)	111 ± 14	116 ± 14	0.05
Office diastolic BP (mmHg)	72 ± 9	75 ± 10	0.69

Mean office SBP increased significantly from 111 ± 14 mmHg at baseline to 116 ± 14 mmHg (*p* = 0.05) at six months, but this was not confirmed by the ABP substudy (Table 2). The mean day and night ABP values for each group are shown in Table 3. The mean nocturnal SBP was higher at 110 ± 6 mmHg in the HIV-positive group at baseline compared to 99 ± 6 mmHg in the control group (*p* < 0.0001). There were no significant differences in age, gender, ethnicity, BMI, CD_4_ count, ART status or office BP between those patients who did and those who did not undego ABP monitoring.

**Table 3:** Mean day and night BP and dipping status in 30 patients with HIV and in 17 control subjects

	*HIV positive*		*HIV negative*	*Baseline BP vs controls (p-value)*
	*Baseline*	*Six months*		
Mean BP (mmHg)				
Daytime SBP	114 ± 10	116 ± 12	114 ± 14	1.00
Daytime DBP	75 ± 12	72 ± 11	73 ± 16	0.63
Night-time SBP	110 ± 6	111 ± 4	99 ± 6	< 0.0001
Night-time DBP	65 ± 8	67 ± 11	60 ± 9	0.05
Non-dipper, *n* (%)	24 (80)	23 (82)	9 (52.9)	–
Dipper, *n* (%)	6 (20)	5 (18)	8 (47.1)	0.05
Total	30	28	17	–

**Fig. 2.**
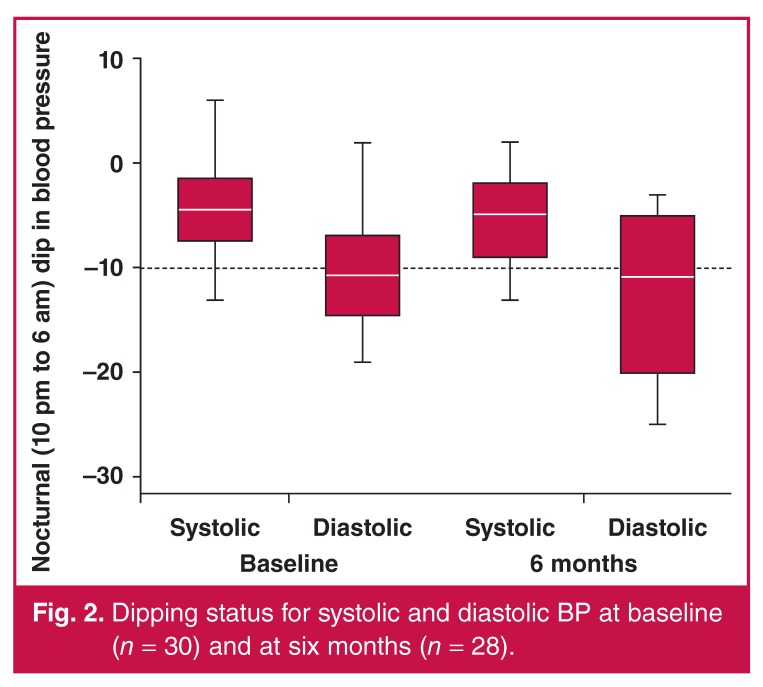
Dipping status for systolic and diastolic BP at baseline (*n* = 30) and at six months (*n* = 28).

The prevalence of non-dipping in HIV-positive patients (Table 3) did not differ at baseline or after six months on ART. Twentyfour of 30 subjects (80%) were non-dippers at baseline and 23 of 28 subjects (82%) (odds ratio = 1.15, *p* = 0.84, 95% CI: 0.31–4.29) were non-dippers at six months (Fig. 2). In the HIV-negative control group, nine of 17 (52.9%) were non-dippers; therefore non-dipping was 3.6 times more likely in HIV-positive patients at baseline than in controls (*p* = 0.05, 95% CI: 0.96–13.13).

## Discussion

This is the first study from Africa, to our knowledge, to have used ABP monitoring to characterise differences in nocturnal blood pressure dipping status between HIV-positive and HIV-negative patients. The study found that: (1) there was a low prevalence of CKD and microalbuminuria in the healthy HIV-positive patients; (2) there was a greater prevalence of non-dipping of nocturnal blood pressure in HIV-positive patients than HIV-negative controls.

Studies from a high-income country found the estimated prevalence of CKD in HIV-infected subjects to be 11 to 15.5%.^23^,^24^ Our study found a lower prevalence of microalbuminuria in HIV-positive patients. In contrast, a study from Johannesburg reported a prevalence of microalbuminuria of 18.5% in their cohort of HIV-positive patients.^25^ A possible explanation for this difference is that their patients were significantly more immunosuppressed (CD_4_ < 200 cells/mm^3^), with a mean CD_4_ count of 130 cells/mm^3^. They also had a high prevalence of co-morbid disease, whereas the patients in our study were all healthy, with a mean CD_4_ count of 239–339 cells/mm^3^.

Microalbuminuria is an important finding in HIV as it may reflect early kidney disease. In a study from KwaZulu-Natal, six of 25 (24%) patients with an eGFR > 60 ml/min/1.73 m^2^ had persistent microalbuminuria and HIV-associated nephropathy (HIVAN) detected on renal biopsy.^26^ This is an isolated study. It is important to note that renal biopsies are not routinely performed in patients with microalbuminuria and normal renal function.

In a large biopsy series from Cape Town, HIVAN presented with nephrotic range proteinuria and impaired renal function.7 Patients not receiving ART had a poor prognosis.^27^,^28^ Microalbuminuria is non-specific and is a marker of inflammation and cardiovascular risk independent of renal function.^29^

In our small study, no patient had a clinically relevant reduction in eGFR (< 60 ml/min/1.73 m^2^), and only one patient had overt macroalbuminuria, which resolved on treatment with ART. This suggests that the approximate prevalence of CKD in an otherwise-healthy HIV population is about 1.6%, considerably lower than previously reported.^23^,^30^ Importantly, a high CD_4_ count and normal creatinine level does not exclude renal disease in HIV.^6^,^7^,^26^

Patients demonstrating proteinuria, who would not normally be eligible for ART due to an elevated CD_4_ count, benefit from timely initiation of ART, which can greatly improve survival rates, with stabilisation of eGFR.^7^,^30^ Therefore screening of patients enrolling into an ART programme, with urine dipsticks or spot urine sampling for proteinuria should be standard practice and could have an impact on the prevalence of HIVAN. This is particularly important in South Africa where, due to the problem of limited access to renal replacement, there is a need for early identification and management of renal disease.

In this study, no cases of hypertension were identified, and there was a small but significant increase in office SBP after six months on ART. However in a subset of patients, ABP monitoring did not confirm these findings. ABP monitoring is the most reliable method of assessing BP and suggests that the effect of ART on BP may be minimal.

In the ABP substudy there was no difference in mean daytime SBP and DBP between patients and controls. However the mean night-time SBP was significantly higher in the HIV group, as was the proportion of non-dippers compared to the control group with similar demographics (BMI, age, gender, socio-economic status). A non-dipping pattern is an established entity, with important clinical implications, and is associated with a higher cardiovascular morbidity and mortality rate.^31^ The high prevalence of non-dippers in the HIV-infected group in this study supports data from Italy and Norway.^9^,^12^

The potential mechanisms underlying the non-dipping phenomenon in HIV-positive patients are uncertain. It does suggest an underlying dysregulation of the cardiovascular system. Chronic infection and arterial inflammation contribute to endothelial dysfunction, which may be further exacerbated by ART.^2^,^3^ In addition, HIV-related endocrinopathies (i.e. hyperaldosteronism and hypercortisolism) and autonomic dysfunction may play a role.^32^,^33^ However, the lack of improvement in dipping status after six months of ART with suppressed viral loads suggests that other mechanisms may also be involved.

Our study has several limitations. Firstly, the sample size for the ABP substudy was small and the nocturnal dipping status between HIV-positive and HIV-negative controls was marginal. Secondly, the short length of follow up (six months) may have been a limitation as the effects of ART on BP and nocturnal dipping may take longer to manifest. Thirdly, a single spot sample was used for establishing microalbuminuria. Also, ABP measurements were conducted during a presumed typical weekday and we could not objectively observe daytime activity and duration of night-time bed rest, which has been shown to affect diurnal BP patterns. Lastly, the HIV-negative ABP-monitoring control group was recruited from another study.

The focus of HIV care in our country remains viral suppression and management of opportunistic infections. Our findings correlate with established evidence linking HIV to increased cardiovascular risk. Young black females are traditionally at low risk for cardiovascular disease. However a non-dipping status in the context of HIV infection could confer a greater risk in this group.

If there are approximately 5.6 million HIV-positive people in South Africa, potentially 4.48 million (80%) are non-dippers. Therefore, as HIV-infected patients are living longer, investigating and addressing the cardiac and metabolic complications related to HIV is becoming more important.
